# Difficulties Facing Junior Physicians and Solutions Toward Delivering End-of-Life Care for Patients with Cancer: A Nationwide Survey in Japan

**DOI:** 10.1089/pmr.2022.0008

**Published:** 2022-10-27

**Authors:** Soichiro Okamoto, Yu Uneno, Natsuki Kawashima, Shunsuke Oyamada, Yusuke Hiratsuka, Keita Tagami, Manabu Muto, Tatsuya Morita

**Affiliations:** ^1^ Medical Corporation Teieikai Chiba Home Care Clinic, Chiba, Japan.; ^2^ Department of Therapeutic Oncology, Graduate School of Medicine, Kyoto University, Kyoto, Japan.; ^3^ Department of Palliative Care, Tsukuba Medical Center, Tsukuba, Japan.; ^4^ Department of Biostatistics, JORTC Data Center, Tokyo, Japan.; ^5^ Department of Palliative Care, Graduate School of Medicine, Tohoku University, Sendai, Japan.; ^6^ Division of Supportive and Palliative Care, Seirei Mikatahara General Hospital, Hamamatsu, Japan.

**Keywords:** end-of-life care, medical education, palliative care, palliative care difficulties scale, physician support

## Abstract

**Background:**

Junior physicians' perceived difficulty in end-of-life care of patients with cancer has not been structurally investigated; therefore, current challenges and solutions in this area remain unknown.

**Objectives:**

To identify some difficulties junior physicians face in delivering end-of-life care for patients with cancer and to clarify the support required to reduce these difficulties.

**Design:**

A nationwide survey was conducted in over 300 institutions selected randomly from 1037 clinical training hospitals in Japan.

**Participants:**

From each of these institutions, two resident physicians of postgraduate year (PGY) 1 or 2, two clinical fellows of PGY 3–5, and an attending physician were requested to respond to the survey.

**Measurements:**

The survey investigated issues regarding end-of-life care using the palliative care difficulties scale with two additional domains (“discussion about end-of-life care” and “death pronouncement”). Items related to potential solutions for alleviating the difficulties as well were investigated.

**Results:**

A total of 198 resident physicians, 134 clinical fellows, and 96 attending physicians responded to the survey (response rate: 33.0%, 22.3%, and 32.0%). The results revealed that junior physicians face difficulties within specific domains of end-of-life care. The most challenging domain comprised communication and end-of-life discussion with patients and family members, symptom alleviation, and death pronouncement. The most favored supportive measure for alleviating these difficulties was mentorship, rather than educational opportunities or resources regarding end-of-life care.

**Conclusion:**

The findings of this study reveal the need for further effort to enrich the mentorship and support systems for junior physicians delivering end-of-life care.

## Introduction

Cancer represents a major global health concern, responsible for 9.6 million deaths every year and accounting for one in six deaths worldwide.^1^ As populations continue to age, the number of cancer-related deaths is estimated to grow to over 16.4 million in 2040, increasing the frequency and importance of the role of health care professionals involved in end-of-life care.^1^ The National Hospice and Palliative Care Organization defines end-of-life care as from the time a patient is diagnosed with a terminal illness that has a life expectancy of less than six months.^2^ End-of-life care requires multidisciplinary care to alleviate various types of distress, including physical, psychological, social, and spiritual burdens.^3–5^ Moreover, health care professionals are expected to interact with patients and their families to discuss the treatment plan, place of care, and wishes for life-prolonging treatment.^6–8^ Accordingly, health care professionals, including physicians, are required to show dedicated and thoughtful behavior and care toward patients and their caregivers.

Physicians face various types of difficulties in end-of-life care, and there are also physician-specific difficulties such as death pronouncement. Of the various types of knowledge, skills, and experience, physicians are expected to acquire early in their clinical training, they reported feeling the considerable conflict and burden in managing dying patients and their families.^9–15^ In addition to the tasks of physician at the end of life, death pronouncement is also a challenging exclusive task of physicians in various countries, including Japan.^16,17^ One qualitative study of the difficulties faced by junior physicians found them aware of the complexity of end-of-life care across various domains, including providing appropriate explanations to patients' families, an understanding of patient needs, theoretical knowledge of end-of-life care, avoidance of conflicts, and time coordination.^10^ Furthermore, it has been established that junior physicians do not receive adequate supervision in many cases despite frequently providing end-of-life care to patients.^12^ Thus, junior physicians might experience considerable burden when they are involved in end-of-life care.

However, to date, investigation into the difficulties faced by junior physicians delivering end-of-life care has been limited; furthermore, the seriousness and priorities of their current challenges and solutions have not been fully clarified. In other words, although several qualitative studies have revealed that junior physicians felt difficulties in end-of-life care, a quantitative survey has not been conducted to investigate the comparison and frequency of the difficulties' characteristics. That knowledge has the potential to further elucidate educational strategies to achieve dissemination of quality end-of-life care practices.

Thus, this study was aimed at identifying the difficulties junior physicians face in end-of-life care and at clarifying methods of helpful support that might reduce these difficulties and strengthen their confidence.

## Materials and Methods

### Study design

This study consisted of a nationwide survey administered to junior physicians in the departments of internal medicine and surgery at government-designated clinical training hospitals. This study was reviewed and approved by the Ethics Committee of Tsukuba Medical Center Hospital. The detailed aims and concept of the survey were explained in the documents given to the participants, and the response of the survey was deemed to be consent to participate.

### Participants

The study participants were enrolled from 300 institutions selected randomly from 1037 government-designated clinical training hospitals in Japan. The list of government-designated clinical training hospitals was obtained from the website of the Ministry of Health, Labor, and Welfare of Japan as of April 2019. Two resident physicians of postgraduate year (PGY) 1 or 2, two clinical fellows of PGY 3–5, and an attending physician at each institution were requested to respond to the survey. Of the two residents and clinical fellows, one each was from internal medicine and the other was a surgeon. The inclusion criteria of the survey were physicians of internal medicine or surgery who worked at government-designated clinical training hospitals and treated patients with cancer. To ensure accuracy of the physicians' responses, we considered the sample size so that the 95% confidence interval would be within ±7.5% of the point estimate of the proportion, which would require responses from a minimum of 171 physicians in each grade.

Two responses each from resident physicians and clinical fellows of 300 facilities were requested. If the response rate was 40%, 240 responses were expected to provide the intended response accuracy. As we thought it was challenging to investigate the status regarding end-of-life care in various clinical scenarios (i.e., emergency medicine and intensive care), this survey was conducted with physicians who routinely treat patients with cancer. No exclusionary criteria were defined.

### Content of the questionnaire

At the beginning of the survey, “end-of-life care” was defined in accordance with previous literature as “whole person care to relieve various burdens, including physical and psychological distress and to improve quality of life for patients and their families whose prognosis is expected to be within six months.”^18^ In addition, the participants were instructed to answer questions about terminally ill patients with cancer. The outline of the questionnaire is described in Supplementary Table S1.

#### Palliative Care Difficulties Scale (PCDS)

Palliative Care Difficulties Scale (PCDS), developed by Nakazawa et al,^19^ was used to investigate the difficulties associated with delivering end-of-life care. The statistical reliability and validity of PCDS were verified, with the scale consisting of five domains comprising 15 items: “communication in multidisciplinary teams”; “communication with the patient and family”; “expert support”; “alleviation of symptoms”; and “community coordination.” Two additional domains were investigated, “discussion about end-of-life care” and “death pronouncement,” comprising six items (Supplementary Table S2). This approach was because, on the basis of the extant literature and our research interest focused on end-of-life care, we thought it necessary to add these two domains to PCDS, considering the wider range of skills and knowledge.^16,17,20,21^ The responses were recorded using a 5-point Likert scale (“strongly agree,” “agree,” “neither agree nor disagree,” “disagree,” and “strongly disagree”), with a higher score indicating a greater sense of difficulty. The internal validity of the “discussion about end-of-life care” and “death pronouncement” domains added in this study was verified using Cronbach's alpha.^22^

#### Support for alleviating the difficulties associated with delivering end-of-life care

We examined items of supportive measures for alleviating the difficulties of junior physicians delivering end-of-life care, on the basis of findings of a comprehensive literature review, focus group discussions (review meetings with six experienced palliative care physicians in 2019), and discussions among researchers.^8–15,23–27^ The criteria for item selection included clarity of the research concept and high clinical importance, and a list of 11 items was developed. The 11 items were rated on a 5-point Likert scale of “not useful,” “somewhat useful,” “useful,” “very useful,” and “essential.” A free text query was prepared by seeking opinions regarding support needs for alleviating difficulties associated with delivering end-of-life care.

### Background information

Background information (e.g., age, gender, and years of clinical experience) ascertained from each of the participants was described in Supplementary Table S1.

### Survey process

The paper-based survey was distributed to the 300 selected institutions in January 2020 using a self-administered, anonymous questionnaire, and a reminder was sent in February. This approach was because we thought only a paper-based survey could reach the target population. The responses were collected by the end of April 2020.

### Statistical analyses

Descriptive statistics were performed where appropriate, including frequency, proportions, and their confidence intervals. The validity of PCDS, including two additional domains, was confirmed using the factor analysis method. In addition, the differences between groups (i.e., resident physicians, clinical fellows, and attending physicians) were tested using Cohen's *D* statistics.^28^ All analyses were conducted using the statistical package R (version 4.0.3). Participants' free comments were qualitatively analyzed using inductive content analysis.^29,30^ Two independent investigators (S.O. and N.K.) reviewed and generated the codes. The emerging codes were compared and discussed with an expert PC physician (Y.U.) to achieve agreement regarding the codes labeled from the data. To ensure rigor and trustworthiness, an experienced investigator (T.M) supervised and examined the consistency of results.

## Results

### Participant background characteristics

A total of 198 resident physicians, 134 clinical fellows, and 96 attending physicians responded to the survey (response rate: 33.0%, 22.3%, and 32.0%, respectively). The background characteristics of the participants are listed in Table 1. The mean age (±SD) of the resident physicians, clinical fellows, and attending physicians was 27.5 ± 2.9, 29.2 ± 2.4, and 52.2 ± 9.1 years, respectively. The number (proportion) of women was 48 (24.4%), 47 (35.1%), and 84 (88%).

**Table 1.** Characteristics of the Participants

	Resident physicians (***N*** = 198)	Clinical fellows (***N*** = 134)	Attending physicians (***N*** = 96)
Age (mean ± SD)	27.5 ± 2.9	29.2 ± 2.4	52.2 ± 9.1
Sex (female), *n* (%)	48 (24.4%)	47 (35.1%)	84 (88.0%)
Clinical experience year (mean ± SD)	1.6 ± 0.5	3.9 ± 0.8	26.5 ± 9.1
Married, *n* (%)	37 (18.7%)	45 (33.6%)	86 (89.6%)
Own family long-term care experience, *n* (%)	24 (12.1%)	13 (9.7%)	40 (41.7%)
Own family bereavement experience, *n* (%)	164 (82.8%)	100 (74.6%)	87 (90.6%)
Religion, *n* (%)	46 (23.2%)	30 (22.4%)	35 (36.5%)
Specialty, *n* (%)			
Gastroenterology	50 (25.3%)	53 (39.6%)	31 (32.3%)
Respiratory	20 (10.1%)	12 (9.0%)	10 (10.4%)
Neurology	14 (7.1%)	7 (5.2%)	5 (5.2%)
Cardiology	13 (6.6%)	9 (6.7%)	1 (1.0%)
General Internal medicine	8 (4.0%)	12 (9.0%)	11 (11.5%)
Clinical Oncology/Hematology	6 (3.0%)	3 (2.2%)	10 (10.4%)
Emergency medicine	5 (2.5%)	5 (3.7%)	1 (1.0%)
Undecided	22 (11.1%)	1 (0.7%)	—
Others	50 (25.3%)	32 (23.9%)	25 (26.0%)
Death pronouncement experience, *n* (%)			
More than 10 cases	20 (10.3%)	74 (55.6%)	—
Interest in palliative care, *n* (%)	174 (88.3%)	125 (93.3%)	89 (92.7%)
Education regarding palliative care, *n* (%)	175 (88.8%)	122 (91.0%)	21 (21.9%)
Clinical training in palliative care, *n* (%)	67 (34.0%)	60 (44.8%)	31 (32.3%)
Attendance of an onsite palliative care seminar, *n* (%)	124 (62.9%)	104 (78.2%)	88 (91.7%)
Support regarding end-of-life care by (as) mentor, *n* (%)	98 (50.8%)	80 (60.6%)	73 (76.0%)
Availability of palliative care consultation, *n* (%)	116 (58.6%)	98 (73.1%)	70 (72.9%)
Opportunity to discuss end-of-life care with other health care professionals, *n* (%)	147 (74.6%)	121 (90.3%)	93 (96.9%)
Involvement of other health care professionals at important meetings with patients and family, *n* (%)	173 (87.8%)	121 (90.3%)	89 (92.7%)
Attendance of death conference, *n* (%)	86 (43.9%)	60 (44.8%)	51 (53.1%)

SD, standard deviation.

### Comparison of end-of-life care difficulties

**FIG. 1.**
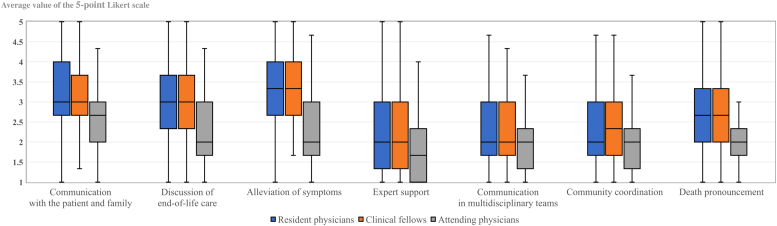
Difficulty status for each domain and the status comparison between junior and attending physicians. Linear bar, range of value; Color bar, Interquartile range; horizontal line, median.

Figure 1 and Table 2 described the difficulty status for each domain, and the status comparison between junior and attending physicians is provided in Table 3. Resident physicians and clinical fellows tended to score higher on “Communication with the patient and family,” “Discussion about end-of-life care,” and “Alleviation of symptoms.” In addition to those domains, “Death pronouncement” also showed considerable differences in difficulties when compared with the case of attending physicians (Cohen's *D* score, 0.57–1.04) (Table 3). The other three domains tended to have lower values with smaller differences than the preceding five domains did when compared with the case of attending physicians.

**Table 2.** Comparison between Palliative Care Difficulties Scale Domains

	Resident physicians (mean [95% CI])	Clinical fellows (mean [95% CI])	Attending physicians (mean [95% CI])
Communication with the patient and family	3.2 (3.07–3.24)	3.1 (2.98–3.26)	2.5 (2.37–2.72)
Discussion about end-of-life care	2.9 (2.84–3.03)	2.9 (2.74–3.05)	2.4 (2.16–2.57)
Alleviation of symptoms	3.3 (3.16–3.36)	3.2 (3.08–3.40)	2.2 (2.03–2.45)
Expert support	2.3 (2.17–2.39)	2.2 (2.03–2.42)	1.8 (1.64–2.01)
Communication in multidisciplinary teams	2.3 (2.21–2.38)	2.3 (2.11–2.44)	2.0 (1.82–2.10)
Community coordination	2.3 (2.24–2.42)	2.3 (2.20–2.54)	2.0 (1.81–2.16)
Death pronouncement	2.6 (2.54–2.73)	2.6 (2.48–2.80)	2.1 (1.88–2.22)

CI, confidence interval.

**Table 3.** Effect Size between Palliative Care Difficulties Scale Domains

	Clinical fellows vs. attending physicians (Cohen's* D *[95% CI])	Resident physicians vs. attending physicians (Cohen's* D *[95% CI])
Communication with the patient and family	0.69 (0.46–0.92)	0.69 (0.42–0.96)
Discussion about end-of-life care	0.6 (0.37–0.83)	0.57 (0.3–0.85)
Alleviation of symptoms	0.97 (0.74–1.21)	1.04 (0.75–1.32)
Expert support	0.43 (0.2–0.65)	0.38 (0.11–0.65)
Communication in multidisciplinary teams	0.39 (0.16–0.62)	0.37 (0.1–0.64)
Community coordination	0.41 (0.19–0.64)	0.41 (0.14–0.68)
Death pronouncement	0.63 (0.41–0.86)	0.67 (0.4–0.94)

### Support to promote confidence in end-of-life care

Table 4 lists the suggested supportive measures for alleviating the difficulties associated with end-of-life care compiled from the responses of the survey participants. Of these potential measures, mentorship from senior physicians was the most helpful form of support (56.1% for resident physicians vs. 57.9% for clinical fellows vs. 62.1% for attending physicians), followed by mental support for junior physicians (44.4% for resident physicians vs. 34.6% for clinical fellows vs. 60.6% for attending physicians). In addition, junior physicians tended to prioritize postgraduate education rather than undergraduate education; however, more attending physicians emphasized undergraduate education.

**Table 4.** Survey Participant Responses Related to Supportive Measures for Alleviating Difficulties in End-of-Life Care

	Resident physicians (%)	Clinical fellows (%)	Attending physicians (%)
More lectures on end-of-life care and symptom relief in pregraduate education.	18.2	17.9	30.2
More clinical practice on end-of-life care and symptom relief in pre-graduate education	23.2	28.4	37.5
More training on communication in pregraduate education	19.7	23.9	43.8
Mandatory clinical rotation training in the palliative care department and palliative care team	31.8	37.3	41.7
Increased opportunities for end-of-life care lectures and workshops through postgraduate education	36.9	25.6	41.1
Enrich self-learning resources related to end-of-life care, such as guidelines and electronic journals.	29.8	23.3	28.4
Attending physicians or mentors to whom younger physicians can easily make consultation, including end-of-life care.	56.1	57.9	62.1
Attending physicians' consideration to make younger physicians experience end-of-life care.	37.9	26.3	38.9
Attending physicians' consideration to observe their end-of-life care practice	38.4	32.3	40
Receive guidance from medical staff such as nurses on the significance of multidisciplinary approach and how to proceed.	34.8	26.3	42.1
Staff or departments where younger physicians can discuss their own mental burden and stress in the training.	44.4	34.6	60.6

### Qualitative analysis of a free text query

In total, 57 (28.9%) resident physicians, 44 (32.8%) clinical fellows, and 38 (39.6%) attending physicians responded to the free text query. Summarized data are presented in Supplementary Tables S2–S5. Five major categories were generated: gaining clinical experience, team-based approach for terminally ill patients, clinical training for end-of-life care, learning opportunities for end-of-life care, and psychological support from senior and peer physicians. Based on typical responses, experiencing and reflecting on many cases were considered important, and so was receiving supervision from mentors and palliative care specialists. Psychological support for junior physicians was also mentioned, and the attending physicians gave their opinions on the quality of mentorship.

## Discussion

Our survey revealed that junior physicians in Japan faced difficulties in specific domains of end-of-life care and suggested potential solutions to improve confidence and skills. The most challenging domains included communication and end-of-life discussion with patients and family members, symptom alleviation, and death pronouncement. The most favored supportive measure to alleviate the difficulties was mentorship rather than off-the-job training opportunities or self-learning resources regarding end-of-life care. These findings highlighted the perceived educational value of attending physicians' behaviors for improving the end-of-life care skills and confidence of junior physicians. Moreover, the findings revealed challenging domains, which were expected to contribute future consideration of their educational strategies.

In this study, the junior physicians tended to have a higher sense of difficulty in the “communication with the patient and family” and “alleviation of symptoms” domains (Fig. 1). Our research also revealed that the difficulties faced by junior physicians were higher than that noted in previous studies.^31–33^ Physicians involved in end-of-life care are required to deal with physical, mental, psychosocial, and spiritual suffering of patients. Although communication and pain/symptom management have been recognized as important areas of competency required of residents delivering end-of-life care, junior physicians might face challenges in treating end-of-life patients and difficulties in their clinical practice because they lack experience and knowledge.^27,34^ Although it has been internationally reported about various types of educational opportunities and resources for learning about symptom alleviation and communication skills, additional strategies to support junior physicians would be needed.^35^

This study also revealed that junior physicians have difficulties regarding “discussion of end-of-life care” and “death pronouncement.” Previous qualitative research indicates that “discussion about end-of-life care” and “death pronouncement,” added as domains in this study, are competencies required for residents and are factors related to posttraumatic stress.^36^ The “discussion about end-of-life care” includes delivering bad news that the prognosis is limited and there is no curative treatment, which is challenging for junior physicians.^37,38^ Death pronouncement also involves both knowledge and technical issues (e.g., how to confirm death), as well as communication before and after death (e.g., how to tell the family that the patient has died, how to understand the family's feelings, and how to offer comfort and encouragement).^39,40^ It might be necessary to develop a systematic training program regarding end-of-life discussions and death pronouncement.^41,42^

The potentially helpful support measures included in this study are (1) more lectures on end-of-life care and symptom alleviation in undergraduate education; (2) additional clinical practice; (3) greater practice in communication with patients and their caregivers; (4) mandatory clinical training in palliative care departments and palliative care teams in postgraduate education; and (5) increased opportunities to attend seminars and workshops on end-of-life care, among others. Although 30%–43% of the attending physicians thought that these undergraduate and postgraduate education programs would be helpful, only 18%–37% of the junior physicians thought they would be helpful. This discrepancy might be due to differences between the attending physicians' expectations of the effectiveness of interventional support measures and the views of junior physicians who have received or are receiving undergraduate and postgraduate education. Further research is needed to understand the reasons for these differences. Moreover, educational interventions on palliative and end-of-life care, as well as palliative care rotations, have been demonstrably effective measures for improving knowledge and self-awareness and beneficial for some junior physicians.^27,34,43^

It has been suggested that early training in palliative care for physicians in training enhances expertise, patient-centered medicine, the psychosocial and spiritual aspects of palliative care, communication, teamwork, and self-awareness.^24^ In addition, in the current study, the highest percentage of respondents indicated that it would be helpful to have the option of a supervisor/mentor with whom they could easily consult on end-of-life care. The residents in this study emphasized the importance of obtaining emotional support. Reportedly, junior physicians might be experiencing emotional distress because they lack supervision from senior physicians and mentors.^24,42–44^ Attending physicians are considered to play a long-term guiding role in personal and professional growth through their involvement with junior physicians as mentors who provide emotional support and encouragement.^45,46^ Although educational interventions and gaining clinical experience are important, it might be necessary to establish a support system that allows residents to safely gain experience in a secure environment.

There are several limitations associated with this study. The first limitation is the possible influence of Japan's unique culture. Asian cultures place more emphasis on family-involved decision making than they do on patient autonomy, which may increase the complexity and difficulty of communication, for instance.^47,48^ The second was external validity. Our survey was targeted at junior physicians involved in residency or fellowship clinical training, not less-experienced mid-career physicians. Although this study did not intend that those less-experienced physicians be included, some of the results might be partially applicable to them. The third was a methodological issue. For instance, we received a low response rate of 28%–32%. This low response rate might indicate that respondents interested in the difficulties of end-of-life care responded willingly and that the results could be overestimated. Biases related to sampling also need to be considered. Random sampling was performed to ensure the representativeness of the facility, but within the facility, the clerical staff was just requested to distribute it to internal medicine and surgeons, and no action was requested to ensure the representativeness of the background characteristics of the physicians.

Female physicians account for less than half of the total number of physicians in Japan; 88% of the attending physicians who responded to this survey were female, which might bias the results. This study was not conducted through e-mail but was paper-based for a purpose that of reaching the study population. Future studies should employ other survey formats (e.g., e-mail surveys) or sampling methods closer to the actual population to eliminate such bias.

## Conclusions

This study revealed that inexperienced junior physicians in Japan faced difficulties in specific domains of end-of-life care and that the enrichment of the mentorship and support system for junior physicians is important. Further effort is warranted to clarify whether mentorship and support systems can relieve the difficulties faced by junior physicians and improve the quality of the end-of-life care they deliver.

## Supplementary Material

Supplemental data

Supplemental data

## Abbreviations Used

PCDS: Palliative Care Difficulties Scale
PGY: postgraduate year
