# Supplementary material for: Difficulties Facing Junior Physicians and Solutions Toward Delivering End-of-Life Care for Patients with Cancer: A Nationwide Survey in Japan
Source: Palliat Med Rep. 2022 Oct 27;3(1):255–63. doi: 10.1089/pmr.2022.0008 (PMC9629909; doi:10.1089/pmr.2022.0008)
Supplement: Supplemental data [file Suppl_TableS1.docx]

Additional Table 1. The outline of the questionnaire

| Background information | |  |
| --- | --- | --- |
|  | age, gender, years of clinical experience, marital status, bereavement experience of close relatives, religious beliefs, major (or planned) field of expertise, interest in palliative care, training history in palliative care, number of end-of-life care patients, and amount of death pronouncement experience. |  |
|  |  |  |
|  |  |  |
|  |  |  |
| Palliative Care Difficulties Scale (PCDS) | |  |
|  | Original version consisted of five domains comprising 15 items “communication in multidisciplinary teams”; “communication with the patient and family”; “expert support”; “alleviation of symptoms”; and “community coordination.”  The two additional domains were “discussion about end-of-life care” and “death pronouncement.” See Additional Table 2 for detail. |  |
|  |  |  |
|  |  |  |
|  |  |  |
|  |  |  |
| Support for alleviating the difficulties associated with delivering end-of-life care | |  |
|  | See Table 3 to find all items |  |
| A free text query was prepared by seeking opinions regarding support needs for alleviating difficulties associated with delivering end-of-life care. | |  |
|  |  |  |
