# Supplementary material for: Difficulties Facing Junior Physicians and Solutions Toward Delivering End-of-Life Care for Patients with Cancer: A Nationwide Survey in Japan
Source: Palliat Med Rep. 2022 Oct 27;3(1):255–63. doi: 10.1089/pmr.2022.0008 (PMC9629909; doi:10.1089/pmr.2022.0008)
Supplement: Supplemental data [file Suppl_TableS2-S5.docx]

| Additional Table 2. The two domains added to the Palliative Care Difficulties Scale and Cronbach’s | | |
| --- | --- | --- |
|  | | Cronbach' α |
| Communication with the patient and family | | 0.80 |
| Alleviation of symptoms | | 0.83 |
| Communication in multidisciplinary teams | | 0.82 |
| Expert support | | 0.83 |
| Community coordination | | 0.83 |
| Discussion about end-of-life care | | 0.80 |
|  | It is difficult to discuss the limited prognosis with a patient and their family. |  |
|  | It is difficult to inform a patient and their family that there are no curative treatments. |  |
|  | It is difficult to discuss the code status with a patient and their family. |  |
| Death pronouncement | | 0.82 |
|  | It is difficult to deliver the death pronouncement. |  |
|  | It is difficult to grasp the feelings of the bereaved family when delivering the death pronouncement. |  |
|  | It is difficult to talk to the bereaved family before and after death pronouncement. |  |

| Additional Table 3. Summarized data of qualitative analysis of resident physicians' responses to a free text query (Overall participants, n= 198; Free text respondents, n=57.) | | |
| --- | --- | --- |
| Major categories | Minor categories | n |
| Gaining clinical experience | |  |
|  | Experiencing several cases of end-of-life care | 9 |
|  | Experiencing difficult cases of end-of-life care | 4 |
|  | Reflecting on cases of end-of-life care | 3 |
|  | Leading the management of end-of-life care | 2 |
| Team-based approach for terminally ill patients | |  |
|  | Team-based approach so as to avoid practicing alone | 12 |
|  | Support from senior physicians | 9 |
|  | Support from palliative care specialists | 6 |
| Clinical training for end-of-life care | |  |
|  | Pre- and post-graduate training opportunities for palliative care | 9 |
|  | Opportunity to observe senior physicians' practice | 6 |
|  | Securing time to deliver end-of-life care | 5 |
|  | Training according to individual interests | 2 |
| Leaning opportunities for end-of-life care | |  |
|  | Systematically learning about palliative care | 11 |
|  | Off-the-job learning opportunities (e.g., simulation workshop) | 6 |
|  | Self-learning tools or opportunities for end-of-life care | 6 |
|  | Learning opportunities for communication | 3 |
| Psychological support from senior physicians | |  |
|  | Psychological support from senior physicians | 5 |
| Others |  |  |
|  | Gaining life experience | 1 |

| Additional Table 4. Summarized data of qualitative analysis of clinical fellows' response to a free text query (Overall participants, n= 134; Free text respondents, n=44.) | | |
| --- | --- | --- |
| Major categories | Minor categories | n |
| Gaining clinical experience | |  |
|  | Experiencing several cases of end-of-life care | 7 |
|  | Experiencing difficult cases of end-of-life care | 1 |
| Team-based approach for terminally ill patients | |  |
|  | Team-based approach so as to avoid practicing alone | 10 |
|  | Support from senior physicians | 12 |
|  | Support from palliative care specialists | 14 |
| Clinical training for end-of-life care | |  |
|  | Pre- and post-graduate training opportunity for palliative care | 10 |
|  | Opportunity to observe senior physicians' practice | 2 |
|  | Securing opportunity to deliver end-of-life care | 8 |
| Leaning opportunities for end-of-life care | |  |
|  | Self-learning tools or opportunities for end-of-life care | 6 |
|  | Learning opportunities for communication | 2 |
| Mental support from senior physicians | |  |
|  | Mental support from senior and peer physicians | 5 |
| Others |  |  |
|  | Social enlightenment regarding palliative care | 2 |
|  | Learning opportunities regarding thanatology | 2 |

| Additional Table 5. Summarized data of qualitative analysis of a free text query from attending physicians (Overall participants, n= 96; Free text respondents, n=38.) | | |
| --- | --- | --- |
| Major categories | Minor categories | n |
| Gaining clinical experience | |  |
|  | Experiencing many cases of end-of-life care | 8 |
|  | Reflecting on cases of end-of-life care | 3 |
|  | Leading the management of end-of-life care | 1 |
| Team-based approach for terminally ill patients | |  |
|  | Support from senior physicians | 5 |
|  | Team-based approach so as to avoid practicing alone | 4 |
|  | Support from palliative care specialists | 4 |
| Clinical training for end-of-life care | |  |
|  | Training according to individual interests | 8 |
|  | Training mentors and developing a reasonable clinical training program | 4 |
|  | Teaching younger physicians to understand the importance of palliative care | 3 |
|  | Teaching younger physicians to understand the importance of patient-centered care | 3 |
|  | Securing opportunity to deliver end-of-life care | 3 |
|  | Pre- and post-graduate training opportunities for palliative care | 1 |
| Learning opportunities for end-of-life care | |  |
|  | Systematically learning about palliative care | 2 |
| Others |  |  |
|  | Religious support | 1 |
|  | Opportunity to learn humanities and sociology | 1 |
|  | Social enlightenment regarding palliative care | 1 |
